# The role of biomarkers in personalized immunotherapy

**DOI:** 10.1186/s40364-022-00378-0

**Published:** 2022-05-18

**Authors:** Kamya Sankar, Jing Christine Ye, Zihai Li, Lei Zheng, Wenru Song, Siwen Hu-Lieskovan

**Affiliations:** 1 Division of Hematology/Oncology, Department of Internal Medicine, University of Michigan, Ann Arbor, MI USA; 2 Rogel Cancer Center, University of Michigan, Ann Arbor, MI USA; 3 Pelotonia Institute for Immuno-Oncology, The Ohio State University, Columbus, OH USA; 4 Johns Hopkins University, Baltimore, MD USA; 5 Kira Pharmaceuticals, Cambridge, MA USA; 6 Division of Medical Oncology, University of Utah, Salt Lake City, UT USA; 7 Huntsman Cancer Institute, Salt Lake City, UT USA

**Keywords:** Biomarkers, Immune checkpoint inhibitors, Immuno-oncology, Combination immunotherapy, Resistance mechanisms

## Abstract

**Background:**

Immune checkpoint inhibitors have revolutionized cancer therapeutic paradigm and substantially improved the survival of patients with advanced malignancies. However, a significant limitation is the wide variability in clinical response.

**Main text:**

Several biomarkers have been evaluated in prior and ongoing clinical trials to investigate their prognostic and predictive role of patient response, nonetheless, most have not been comprehensively incorporated into clinical practice. We reviewed published data regarding biomarkers that have been approved by the United States Food and Drug Administration as well as experimental tissue and peripheral blood biomarkers currently under investigation. We further discuss the role of current biomarkers to predict response and response to immune checkpoint inhibitors and the promise of combination biomarker strategies. Finally, we discuss ideal biomarker characteristics, and novel platforms for clinical trial design including enrichment and stratification strategies, all of which are exciting and dynamic to advance the field of precision immuno-oncology.

**Conclusion:**

Incorporation and standardization of strategies to guide selection of combination biomarker approaches will facilitate expansion of the clinical benefit of immune checkpoint inhibitor therapy to appropriate subsets of cancer patients.

## The current landscape of cancer immunotherapy

Over the last decade, immune checkpoint inhibitors (ICI) have transformed the landscape of treating advanced malignancies, however, response rates remain widely variable. Hence, biomarkers with high sensitivity and specificity are required to identify sub-populations who are most or least likely to elicit a sustained response. Many patients with solid and hematologic malignancies have the potential to respond to ICI given that tumors are different from self and anti-tumor immune responses is anticipated. However, intrinsic features of the tumor, tumor microenvironment (TME) and defects in the host’s innate and adaptive immune mechanisms may compromise an effective anti-tumor immune response and impact the host’s response to ICI. To overcome immuno-resistance, rational combinatorial approaches have been evaluated and implemented into clinical practice for various tumor types (e.g., checkpoint inhibitors in combination with chemotherapy, targeted therapy or other immune-modulatory agents). The future of successfully treating cancers with single-agent or combinatorial ICI strategies will depend on advances in the identification and standardization of predictive biomarkers. An ideal biomarker will be multifaceted in identifying patients who are most likely to derive benefit from ICI while also limiting exposure and toxicity. In this review, we aim to describe the current practice of utilizing biomarkers in ICI monotherapy, dual biomarker approaches in combinatorial ICI therapies, and emerging technologies which may represent the future of biomarker development in the era of cancer immunotherapy. This review will include current state of biomarkers for immunotherapy, some of which were updated at the 2020 China Immuno-Oncology (IO) Workshop co-organized by the Chinese American Hematologist and Oncologist Network (CAHON), the China National Medical Product Administration (NMPA) and Tsinghua University.

## The role of biomarkers in the current era of single-agent ICI therapy

Better understanding of the TME and mechanisms of host immune evasion has led to development of various ICIs, which are active across solid and hematologic malignancies. The first ICI approved by the U.S. Food and Drug administration (FDA) was ipilimumab (anti-cytolytic T lymphocyte Antigen 4 (CTLA-4) therapy) in 2011 after it was shown to prolong overall survival (OS) in advanced melanoma [1]. Since then, seven additional agents have been approved with the incorporation of blocking programmed cell death protein 1 (PD1) / PD-Ligand 1 (PD-L1) pathway [2]. Biomarkers to predict response to ICI in advanced malignancies are being extensively studied and some have been clinically validated. Biomarkers may be detected and measured in the tumor tissue or in the peripheral blood.

Herein, we summarize the current FDA approved tissue biomarkers for solid malignancies including PD-L1, tumor mutational burden (TMB), and microsatellite instability (MSI). We will discuss exploratory tissue biomarkers such as tumor gene expression profiling (GEP), multiplex immunohistochemistry (IHC) and immunofluorescence (IF), tumor infiltrating lymphocytes (TILs), immunoscore, T cell receptor (TCR) diversity, and microbiome, as well as cellular and soluble peripheral blood biomarkers.

### Tissue biomarkers

#### Current U.S. FDA approved tissue biomarkers

**Fig. 1:**
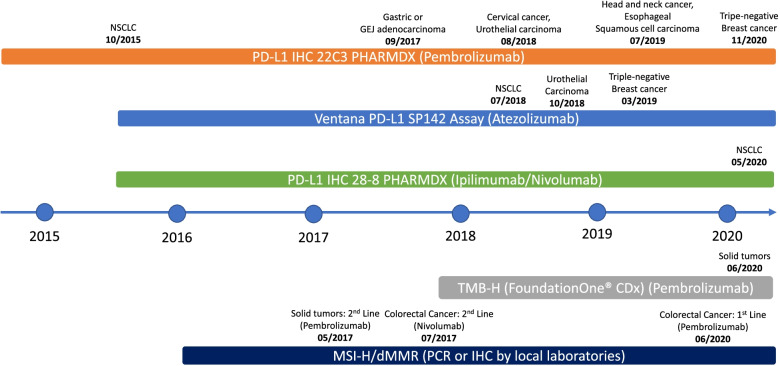
Timeline of biomarker test approvals by the U.S. Food and Drug Administration

Timeline for the approval of biomarkers in clinic application is described in Fig. 1.

##### PD-L1

PD1 is expressed on activated TILs in various tumors, while PD ligands (PD-L1 and PD-L2) are commonly upregulated on tumor cell surfaces [3, 4]. Forced expression of PD-L1 on the surface of mouse tumor cells inhibits local anti-tumoral T cell mediated responses [5, 6], which forms the basis of PD1 pathway blockade in enhancing antitumor function. PD-L1 expression on tumor cells or TILs determined by IHC has become a commonly used biomarker for selecting patients for ICI therapy across tumor types [7, 8].

In The Blueprint PD-L1 Assay Comparison Project, three different PD-L1 assays demonstrated correlation with PD-L1 score and objective response for patients treated with ICI in certain tumor types: PD-L1 IHC 22C3 pharmDx, PD-L1 IHC 28–8 pharmDx, and VENTANA PD-L1 (SP142) [9]. These assays showed high concordance among pathologists for determination of PD-L1 expression on tumor cells; concordance of PD-L1 expression on immune cells was also demonstrated, albeit with greater variability. The two methods to score PD-L1 expression using the PD-L1 IHC 22C3 or 28–8 pharmDx assay are measurement of tumor proportion score (TPS) and combined positive score (CPS) [10]. PD-L1 expression on tumor cells assessed via TPS is a well validated biomarker in non-small cell lung cancer (NSCLC) [11, 12]. The CPS method was developed to aid in selection of patients with urothelial cancer, gastric/gastroesophageal adenocarcinoma, triple-negative breast cancer, and ovarian cancer who would benefit from pembrolizumab [13]. The VENTANA SP142 assay, on the other hand, uses the percentage of PD-L1 positive immune cells to determine PD-L1 expression on TILs: IC0 (< 1%), IC1 (≥ 1% but < 5%), and IC2/3 (≥ 5%).

It is important to note that PD-L1 is an imperfect biomarker by itself, as its expression can be triggered by active immune response (i.e., patients with a negative baseline PD-L1 stain might still respond to ICI). Further, tumors with high PD-L1 expression may be resistant to treatment. In fact, a study of 45 FDA approvals of ICI from 2011 to 2019 showed that PD-L1 was only predictive in 28.9% of cases and was either not predictive (53.3%) or not tested (17.8%) in the remaining cases [14]. Only 9 of the approvals were linked to a specific PD-L1 threshold and companion diagnostic assay, with variable thresholds both within and across tumor types using several different assays, suggesting that PD-L1 testing has certain limitations which must be considered in clinical decision making. Therefore, various biomarkers predictive of ICI efficacy independent of PD-L1 status are being evaluated, many of which are described below.

##### MSI and TMB

MSI results from a defective DNA mismatch repair (dMMR) system which leads to clusters of thousands of mutations along microsatellite regions [15]. Tumors with deficient MMR (dMMR) or high MSI (MSI-H) have increased mutational burden which leads to infiltration of T cells in the TME, leading to improved response with anti-PD1/PD-L1 therapies [16]. Furthermore, a higher TMB correlates with a greater probability of displaying neoantigens on the human leukocyte antigen (HLA) molecules of tumor cell surface, eliciting CD8^+^ T cell dependent immune responses and tumor cell lysis [17, 18]. Importantly, TMB as a continuous variable does not have a linear correlation with OS [19], whereas PD-L1 expression has correlated with OS in advanced NSCLC patients [20]. MSI or MMR is tested using multiplex immunohistochemistry or molecular-based tests including polymerase chain reaction (PCR) and next-generation sequencing (NGS) [21]. TMB can be evaluated by whole exome sequencing (WES) or NGS gene panel assays, both of which have been used in clinical trials to correlate with response to ICI in melanoma [22], NSCLC, urothelial carcinoma [23, 24], and colorectal cancer [24]. In a pooled analysis of patients with various cancers treated with anti-PD1/PD-L1 therapies, patients with high TMB had significantly improved progression-free survival as compared to patients with low TMB [25].

In 2017, the U.S. FDA granted its first tissue/site-agnostic approval to pembrolizumab for patients with advanced cancers harboring MSI-H/dMMR who have progressed on prior therapies with no further satisfactory treatment options [26]. Pembrolizumab has recently been approved in the first-line setting for patients with unresectable or metastatic MSI-H or dMMR colorectal cancer [27, 28] and for adult and pediatric patients with unresectable or metastatic TMB-H (≥ 10 mutations/megabase) solid tumors who have progressed on prior therapies with no acceptable alternatives [29]. These approvals are summarized in Fig. 1.

#### Experimental tissue biomarkers

##### TILs/immunoscore, Tumor GEP, multiplex IHC and IF, HLA and TCR diversity

*TILs and Immunoscore*

CTLA-4 or PD1 blockade unleashes cytotoxic T cell activity against the tumor. The density and location of TILs within the TME can predict response to ICI [30]. For example, the increased number of immunogenic peptides in MSI-H tumors is paralleled by an increase in TILs and higher PD-1 expression [31]. As another example, an “immune inflamed” phenotype is characterized by infiltration of CD4^+^ and CD8^+^ T cells in the tumor parenchyma [32] and has been correlated with increased OS across tumor types [30]. “Immunoscore” was determined by quantification of cytotoxic and memory T cell populations within the tumor core and invasive margins and has been shown to be a prognostic marker in colorectal cancer independent of staging [33]. Immunoscore is now being studied as a marker of response to ICI across tumor types. In addition, the immunophenotype of TILs, for example, increased TIL co-expression of PD-1 and CTLA-4 has been correlated with better response to ICI and longer progression-free survival (PFS) [34]. An important clinical limitation of the Immunoscore and immunophenotyping of TILs to predict response to ICI is the infeasibility of obtaining tissue biopsy at various time points during treatment and progression. Furthermore, these techniques do not differentiate T cell clones capable of targeting tumor-associated antigens, thus limiting specificity.

*Tumor GEP*

 GEP is a comprehensive approach to assess response to ICI using high-throughput tests to analyze immunologic transcriptomic patterns which predict sensitivity or resistance to ICI. Interferon-γ (IFN-γ) and related gene signatures have been assessed [35]. IFN-γ is a key cytokine secreted by various immune cells which stimulates both the innate and adaptive immune response, but simultaneously produces feedback inhibition of anti-tumoral immunity via cross-talk with the PD-1 axis and other key immunosuppressive molecules within the TME [36–38]. A 10-gene IFN-γ signature panel, and subsequently a 28-gene panel in patients with metastatic melanoma receiving anti-PD1 ICI was shown to correlate with improved response, PFS, and OS in patients across 9 tumor types receiving PD-L1 blockade [39, 40]. The digitalization of gene expression analysis overcomes the challenges of inter-laboratory variability and observer bias with other techniques such as PD-L1 expression. One limitation of GEP is the dependence of the algorithm for each gene signature on the individual therapy itself, i.e., IFN-γ transcriptomic patterns may correlate only with response to therapy of directly related targets such as downstream PD1/PD-L1 inhibition [35]. Other important limitations include the inability to elucidate the cellular source of gene expression, cellular co-expression, and geographical relationships of cells within the TME. Single cell RNA sequencing discussed in Sect. 6.0 can alternatively provide a higher resolution of cellular expression patterns and differences within the TME.

*Multiplex(m) IHC and IF*

 mIHC/IF allow for simultaneous visualization of multiple proteins on the same tissue section. mIHC/IF techniques provide information regarding spatial relationships and cellular co-expression of multiple markers. The spatial density of PD1/PD-L1 measured by mIHC was found to be predictive of PD1 blockade in patients with metastatic melanoma [41] and merkel cell carcinoma [42]. Recently, Taube *et* al. reported in a systematic review that mIHC/IF assays outperformed other biomarkers including PD-L1 IHC, TMB, and GEP. Additionally, the authors concluded that TMB and mIHC/IF assays may have an additive value in predicting response to PD1 blockade [43].

*HLA*

 Previously, studies have shown that down-regulation of HLA class I antigen peptide complexes by tumor cells is a mechanism of immune escape and often associated with poor prognosis in cancer [44]. Recently, others have investigated whether decreased or absence of HLA molecules and/or defects in the antigen-presenting machinery molecules might predict response to ICI. In a retrospective analysis of two different trials of patients with melanoma treated with anti-CTLA-4 ± anti-PD1 ICI, HLA I expression was a reliable marker of response to anti-CTLA-4 ICI, but not to anti-PD1 ICI [45]. Though HLA I and II antigen expression contributes to tumor cell recognition, their role as predictive biomarkers for ICI remains unknown and would require prospective clinical studies to confirm. Furthermore, understanding of pathways leading to restored HLA expression could be utilized to conceive new combinatorial therapeutic strategies [46].

##### Microbiome

 The gastrointestinal microbiota is important for anti-tumor immunity with many functions on both adaptive and innate immunity. Pre-clinical models have suggested beneficiary effects of a healthy microbiota in mouse models treated with immunotherapy. Clinically, though increased *Bacteroides* in ICI non-responders and *Akkermansia* in ICI responders [47–49] has been reported, most studies have yielded mixed data regarding the predictive role of microbiome composition. Another area of study is the alteration of microbiota to improve ICI response by way of fecal microbiota transplantation, transfer of specific bacteria or beneficial microbial products and lastly, changes in diet. A landmark study in melanoma showed a high-quality diet rich in whole grains to positively correlate with ICI response [50]. Further research is needed to identify the correlative role of microbiota composition with ICI response or toxicity and moreover to validate the effects of microbiota alteration.

### Exploratory peripheral blood-based biomarkers in study

Peripheral blood-based biomarkers are attractive given their non-invasive nature. Both cellular and soluble peripheral blood biomarkers have been studied including immunophenotype of circulating peripheral lymphocytes, tumor-associated neoantigen specific T cells, inflammatory cytokines, and tumor-specific antibodies. To date, there are no U.S. FDA approved peripheral blood-based biomarkers to assess ICI response.

#### Cellular peripheral blood biomarkers

##### Peripheral PD1^+^ CD8^+^ T cells

It has been hypothesized that monitoring of T cell activation in the peripheral blood can predict response to PD1 blockade. In NSCLC patients receiving anti-PD1 ICI, the early rise in Ki-67^+^ PD1^+^ CD8^+^ T cells correlated with clinical benefit [51]. Similar findings were noted in patients with thymic epithelial tumors who received pembrolizumab [52]. Gros *et* al. demonstrated that neoantigen-specific lymphocytes were preferentially enriched in the CD8^+^PD1^+hi^ or CD4^+^PD1^+hi^ subsets, but not in the corresponding bulk or PD1 fractions in patients with gastrointestinal cancers. [53]. Peripheral T cell immunophenotyping warrants further investigating in order to understand of the peripheral systemic impact of ICI on different immune cells and potential correlation with clinical outcome.

##### Tumor antigen-specific peripheral T cells

Tumor antigen-specific T cells, specifically neoantigen-specific T cells, are gaining increasing interest in being viewed as important immunotherapy effectors. Peng *et* al. have reported a sensitive method to recover antigen-specific T cells, where the detected number of neoantigen-specific T cells strongly correlated with treatment response of melanoma patients receiving PD1 blockade [54]. Similarly, Yuan *et* al. showed that in a subset of melanoma patients who express NY-ESO-1, those who had measurable NY-ESO-1-specific CD4^+^ and CD8^+^ T cell responses experienced more frequent clinical benefit to anti-CTLA-4 ICI and a significant survival benefit as compared to patients with an undetectable specific T cell response [55]. Neoantigen specific T cell response were detected in clinical responders with advanced melanoma or NSCLC treated with ICI [56, 57]. Furthermore, neoantigen-specific T cells are a highly immunogenic target for personalized vaccination. Cancer vaccines which target tumor-associated or tumor-specific antigens can achieve chronic therapeutic response because of immunologic memory. Though several challenges, including variable tumor antigens and relatively low immune response, must be overcome to translate vaccines into effective anti-cancer therapies, many are currently being evaluated in various solid tumors [58–61].

#### Soluble Peripheral blood biomarkers

##### *Soluble PD-1 and PD-*L1 Both PD-1 and PD-L1 have soluble forms (sPD1 and sPD-L1) in the peripheral blood and increased levels as measured by ELISA may correspond with ICI response [62]

Lower levels of sPD1 and sPD-L1 may correlate with longer survival in several malignancies [63], while increased post-treatment sPD1 may correlate with favorable response to ICI [62, 64]. Additionally, the magnitudes of the increase in circulating PD-L1 expression on the surface of exosomes released by metastatic melanomas during early stages of treatment were found to be an indicator of the adaptive response of the tumor cells to T cell reinvigoration, therefore stratifying clinical responders from non-responders [65]. As sPD1 retains its ability to bind ligands and hence disrupt the PD1 axis, preclinical models have studied its role as a therapeutic target. However, the lack of standardization for measurement of sPD1 and sPD-L1 in the blood is a significant limitation. Clinical studies in large cohorts of patients will be needed to validate sPD1 clinically and explore its role as an anti-cancer therapeutic target [66].

##### Circulating tumor DNA

Over the last several years, various methods of detecting circulating tumor DNA (ctDNA) have been validated across tumor types to detect minimal residual disease [67, 68] and to predict response to ICI [69]. In patients with advanced NSCLC receiving anti-PD1/PD-L1 ICI, > 50% ctDNA mutant allele fraction decrease from baseline was associated with radiographic response and superior PFS and OS [70]. Recently, a personalized multiplex-PCR NGS assay by Natera Signatera® has been studied in its role to predict ICI response. The Natera® ctDNA assay uses paired tumor and peripheral blood WES to identify 16 tumor-specific variants [68, 71] which are then detected in the peripheral blood through treatment. In patients with advanced solid tumors treated with pembrolizumab, an early decrease in mean ctDNA concentration and on-treatment clearance of ctDNA are highly correlated with improved OS, independent of tumor type, TMB, or PD-L1 status [72]. Hsu *et* al. reported similar results in patients with unresectable hepatocellular carcinoma receiving atezolizumab plus bevacizumab where ctDNA clearance during treatment correlated with improved PFS [69]. ctDNA monitoring using this method has also been shown to detect minimal residual disease after curative intent treatment in order to identify those who will benefit from adjuvant ICI in patients with high-risk muscle invasive bladder cancer [73] and to track tumor progression after curative-intent surgery and adjuvant chemotherapy/ICI treatment in patients with triple-negative breast cancer [74]. These studies highlight the potential for a broad clinical utility for ctDNA-based surveillance in patients treated with ICI which could be generalizable across cancer types. However further prospective larger cohort studies are required prior to incorporation of ctDNA monitoring into clinical practice for patients with advanced malignancies receiving ICI.

##### *Inflammatory cytokines*. Cytokines play a critical role in activation of host immunomodulation

Through activation of immune cells, inflammatory cytokines typically increase after PD1/PD-L1 axis blockade; additionally, various cytokines have been shown to induce PD-L1 expression in tumor cells. Measurement of several inflammatory blood cytokines including IFN-γ, IL-6, IL-8, IL-11, and IL-2 has been evaluated across tumor types in patients receiving ICI [75]. However, various factors including tumor burden, presence of brain metastases, and co-existing conditions such as stress or infection may affect the levels of inflammatory cytokines in peripheral blood limiting their sensitivity and specificity as predictive biomarkers [76].

##### Neutrophil-to-lymphocyte ratio

Low absolute lymphocyte count has been previously established as a marker of poor prognosis across cancer types, and recently has been shown to be associated with poor response to immunotherapy [77]. Additionally, neutrophil-to-lymphocyte ratio (NLR), which can represent the balance between pro-tumoral inflammatory status and anti-tumoral response, has been evaluated for predictive and prognostic value in patients receiving ICI. In a retrospective study of 1714 patients across 16 cancer types, benefit from ICI was significantly higher for patients with NLR low/TMB high as compared to the NLR high/TMB low group [78]. These findings are supported by a recent study of patients with stage III NSCLC treated with or without consolidation immunotherapy, where pre-treatment higher NLR was associated with inferior PFS in both groups with a greater effect in the group of patients treated with ICI, suggesting that pre-treatment NLR may be a predictive biomarker of ICI benefit [79].

### Challenges and limitations of current biomarkers to predict response to immune checkpoint inhibitors

Biomarkers to predict response is critical for the success of ICI therapy, however, we are facing many challenges in clinical development and implementation into standard clinical practice. These challenges include intra-tumoral and inter-tumoral heterogeneity, variability in host immunity, complexity of interactions between tumor and immune cells in the tumor milieu, and evolution of the cancer through treatment [35]. Within the primary tumor itself, certain sub-clones may not be accurately represented by the initial biopsy, and hence, biomarker analysis on pre-treatment tumor tissue may be biased against potentially resistant sub-clones. Sites of distant metastases may also contain various sub-clones of the tumor presenting a discordance between initial biomarker analysis and response to ICI therapy. Furthermore, the interplay of tumor and immune cells of the surrounding TME plays a significant role in amplification or suppression of the tumor-induced immune response. Hence, a biomarker representing the tumor cell alone may be an insufficient illustration of the potential for enhanced immune response with ICI. Importantly, the ability of a host to surmount an anti-tumoral immune response can be dependent on polymorphisms of HLA alleles and variability in the recombination and expression of the T cell receptor. Components of the antigen presenting machinery are challenging to incorporate into any single biomarker assay. Additionally, the cancer can evolve throughout treatment course and therefore resistant clones may not be identified by biomarkers at baseline. Finally, laboratory tests used in biomarker investigation have large variability in pre-analytic processing, diagnosis, and clinical interpretation. Even for PD-L1 expression by IHC, multiple tests are available and are not all interchangeable [80]. These limitations exemplify the challenge in implementing many of the assays mentioned into clinical practice. Newer technologies are emerging to address some of these challenges, though further validation and standardization of these techniques is critical for reproducibility [35].

### Complexity of resistance mechanisms to immune checkpoint inhibitors and strategies to overcome resistance

Resistance mechanisms to ICI may involve tumor intrinsic or extrinsic mechanisms, and these may be primary or acquired over time.

Tumor intrinsic resistance mechanisms include absence of tumor antigens leading to incomplete T cell recognition [81] or alteration of antigen presenting cell machinery [82]. Adaptive resistance can be from loss of T cell function, lack of T cell recognition by downregulation of antigen presentation and development of escape variants in the cancer. For example, the absence of surface expression of HLA class I or deficiency of HLA class I folding and transport to cell surface leads to lack of CD8^+^ T cell recognition and has been documented in acquired resistance to PD1 blockade in melanoma [83]. Alternatively, chronic antigen exposure may lead to precursor memory T cell exhaustion with eventual deletion and lack of memory formation [84]. Immunoediting is a mechanism whereby tumor cells can downregulate neoantigen expression leading to acquired resistance to ICI [85, 86]. In relapsed NSCLC tumors after ICI therapy, loss of neoantigens has been described [87]. Furthermore, tumor-specific T cells which are activated by checkpoint blockade primarily recognize mutational neoantigens [88]; thus, mutations, deletions or epigenetic changes which would lead to loss of expression of mutational neoantigens can all lead to acquired resistance to ICI.

Tumor extrinsic resistance mechanisms to ICI involve other components of the TME, which include T regulatory cells, myeloid derived suppressor cells (MDSCs) and tumor associated macrophages (TAMs), all of which play an important role in immune evasion [89, 90]. Immunosuppressive cytokines are often released by the tumor or TAMs for local suppression of anti-tumoral responses. For example, TGF-β plays an important role in angiogenesis and immunosuppression by stimulating T regulatory cells.

In an effort to overcome these resistance mechanisms, combination ICI strategies have been evaluated to transform immunologically “cold” tumors into “hot” tumors [91], enhance endogenous T cell function [81], or adoptively transfer antigen-specific T cells, etc. These strategies have been described in detail elsewhere [92]. Combination approaches currently in clinical development include increasing the neoantigen quantity (e.g., chemotherapy, radiotherapy and epigenetic modulation mechanisms), altering the neoantigen quality (e.g., neoantigen vaccine, tumor-associated antigen vaccine), and improving antigen presentation and/or T-cell priming (e.g., DC vaccine, oncolytic virus, anti-CTLA4 ICI, chemotherapy, and targeted therapies). Combinations with antibodies which enhance immunostimulatory targets or deplete immunosuppressive T regulatory cells in order to increase T effector cell function include anti-GITR antibody, anti-41BB antibody, anti-OX40 antibody, and anti-ICOS antibody [93–95]. Another strategy is the combination with inhibitors of immunosuppressive molecules in the TME (e.g., IDO inhibitor, CSF1R inhibitor, Adenosine R inhibitor, TGFβ inhibitor, VEGF inhibitor, and PI3K inhibitors). Response to each of these strategies is dependent on the tumor biology and host immunity, and importantly the primary mechanism by which host immune evasion occurs. Identifying resistant mechanisms to single agent ICI therapy with use of novel biomarkers is crucial to developing effective combinatorial strategies. Ideal biomarkers will have the ability to identify a suitable combinatorial approach for each patient.

### Biomarker strategies in combination therapies

**Fig. 2:**
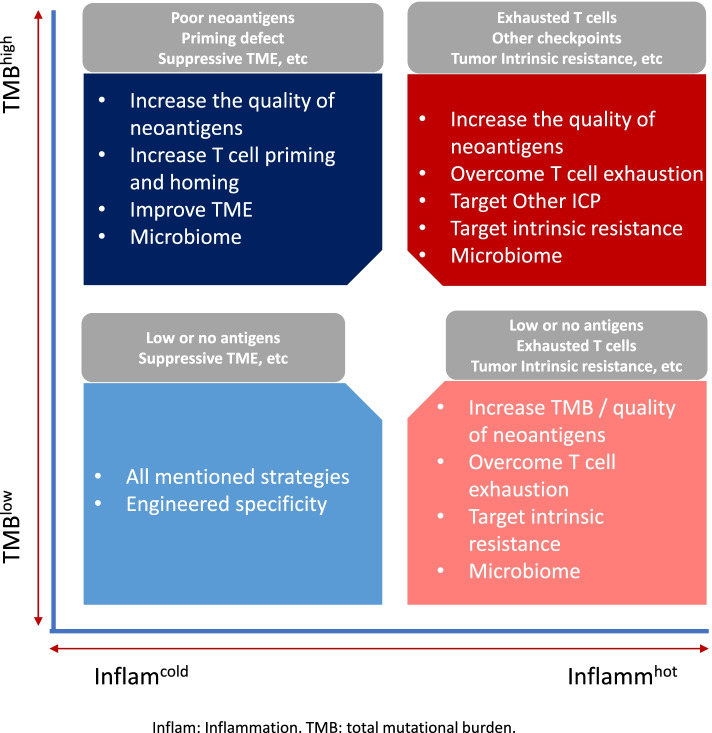
Using dual biomarker approaches to group potential resistant mechanisms for effective combination strategies

Biomarker identification to inform which patient might benefit from combination ICI strategies remains an area of significant interest. Certain biomarkers such as PD-L1 and GEP are inflammatory biomarkers and can characterize an inflamed TME (i.e., “cold” vs. “hot”). Others such as TMB and MSI are related to the tumor immunogenicity (i.e., TMB “high” vs. TMB “low”). Inflammatory and immunogenicity biomarkers are shown to be independent from each other but can be complimentary to help elucidate the potential defects of anti-tumor immune response, therefore guides combination strategy selection. Figure 2 demonstrates how combination biomarker approaches can highlight potential mechanisms of immune resistance including intrinsic signaling and extrinsic host factors in the tumor microenvironment (i.e., TMB^low^Inflam^cold^, etc.). To characterize each of these TME states in the clinical setting will be crucial in order to select appropriate combinatorial approaches that will benefit each patient.

#### PD-L1 in combination with TMB

The predictive power of PD-L1 in combination with TMB was studied in 759 patients with advanced NSCLC who were treated with either anti-PD-L1 ICI alone or in combination with anti-CTLA-4 ICI. TMB was determined by NGS and was compared to PD-L1 expression. While TMB and PD-L1 were independent predictors of ICI efficacy and did not correlate with each other, those with high TMB and PD-L1 ≥ 1% had the highest durable clinical benefit rate [96]. Finally, in a large meta-analysis of 14,395 patients with advanced NSCLC receiving ICI, the combination of PD-L1 and TMB was associated with increased power to predict OS, which was further improved with incorporation of CD8^+^ TILs [97].

#### PD-L1 in combination with CD8^+^TILs

The product of PD-L1^+^ cell and CD8^+^ TILs densities has been studied in baseline tumor biopsies of patients who received durvalumab for advanced cancers in a phase I/II clinical trial. The proportion of CD8^+^ TILs and PD-L1 expression as measured by IHC was used to create a CD8^+^xPD-L1 signature. For those who received durvalumab, a high CD8^+^xPD-L1 signature was associated with a significantly higher OS [98].

#### TMB in combination with GEP

The relationship between TMB and GEP was evaluated by Cristescu et al., in a study involving > 300 patient samples across 22 solid tumor types from four clinical trials. Patients were stratified into four biomarker-defined clinical response groups based on predefined cutoffs for TMB and GEP. The patients with GEP^hi^TMB^hi^ had the highest objective response rates and the longest PFS. Response was moderate in those with GEP^hi^TMB^lo^ and GEP^lo^TMB^hi^ and reduced or absent in those with GEP^lo^TMB^lo^. These groups were then used to guide transcriptome and exome analyses of tumors in a large molecular database of 6384 tumors, where gene enrichment analysis was used to categorize tumors into discrete subgroups (e.g., proliferative, vascular, myeloid and stromal) that exhibited patterns of potentially targetable biology to enhance clinical response [99]. Similar strategies of stratifying patients by combination biomarkers to elucidate the underlying patterns of tumor immunobiology / resistant mechanisms, will enable biology-driven personalization of treatment regimens and advance the field of precision immuno-oncology.

### Emerging novel biomarker technologies: single-cell RNA sequencing (scRNA-seq)

Advances in single-cell RNA sequencing (scRNA-seq) have allowed for comprehensive analysis of the immune system and characterization of tumor heterogeneity through cancer evolution [100–103]. Many of the previously defined technologies are limited by analysis on the most abundant cancer cells, which can mask the profiles of rare cell populations which play key functional roles. Conversely, high throughput scRNA-seq has the potential to reveal the high complexity and diversity of immune infiltrating cells in tumors. For example, the individual transcriptomes of 16,291 individual immune cells from 48 melanoma tumor samples showed that a single transcription factor, *TCF7* visualized within CD8^+^ T cells predicted positive clinical outcomes in an independent cohort of ICI-treated patients. Conversely, cells expressing exhausted or dysfunctional signatures were associated with ICI resistance [104]. Recently, the functional role of non-immune cells in predicting response to ICI was explored by Dominguez *et* al., who identified a population of carcinoma-associated fibroblasts (CAF) that are programmed by TGF-β and express LRRC15 protein in > 80,000 single cells from 22 patients with pancreatic ductal carcinoma. ICI trials comprising of > 600 patients across six cancer types revealed that elevated levels of LRRC15^+^ CAF correlated with poor response to anti-PD1 ICI [105].

scRNA-seq provides transcriptomic signature profiles on several cell types (i.e., tumor cells, immune cells, hematopoietic cells, and non-immune cells), all of which may have an impact on response or resistance to ICI. The future of scRNA-seq will involve combination of other -*omic* techniques as well as delineation of the spatial interaction between different groups of cells. One major limitation in using scRNA-seq to assess ICI response is that analysis requires cell isolation from their environment, making it challenging to assess connections between cells of different compartments which may be overcome by incorporating spatial transcriptomic techniques.

### Incorporation of biomarkers into prospective clinical trials

**Fig. 3:**
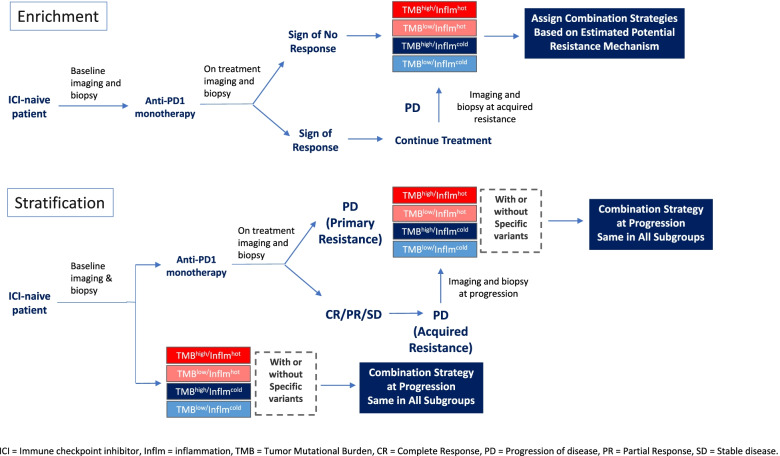
The incorporation of prospective biomarkers in immunotherapy clinical trials

Biomarkers can play an important role with both prognostic and predictive values. How can we integrate biomarkers into clinical trial designs in the era of immunotherapy? A biomarker enrichment strategy has recently gained popularity for efficacy and safety assessments. Biomarkers have been applied to select the appropriate study patient population to either increase the rate of response or decrease toxicity. For example, when appropriate biomarkers are incorporated to select patients who receive frontline anti-PD1 monotherapy, treatment can further be tailored to responders versus non-responders, i.e., switching to combination regimens in the non-responder cohort based on resistance mechanisms determined by selected biomarkers, or continuing the same treatment in the responder cohort. A biomarker stratification strategy is another novel approach where response/clinical benefit is determined in each biological group with distinct resistant mechanisms based on the selected biomarkers, such as TMB and/or inflammation markers. These two strategies for innovative clinical trial design are described in Fig. 3.

It is still a great challenge to identify effective biomarkers and clinical trial design is often complex [106]. NCI Investigational Drug Steering Committee (IDSC) created a Biomarker Task Force who provided recommendations for investigators developing clinical trials with biomarker studies for scientific rationale, assay criteria, trial design and analysis [107]. The FDA also provided guidance for enrichment strategies [108], which is encouraging with its increasing utilization. In addition, unprecedentedly and rapidly emerging biomarker technologies will advance and facilitate these applications in next generation clinical trials.

## Conclusions

We have summarized the role and challenges of currently approved and exploratory tissue and peripheral blood biomarkers in use for predicting response to ICI. While ICI has improved survival and outcome of patients with advanced malignancies, complex resistance mechanisms limit clinical efficacy calling for more accurate prognostic and predictive biomarkers. Combination ICI strategies are being developed to overcome resistance and further improve effectiveness of immunotherapy, and emerging technology allows us to better characterize TME and identify novel biomarkers. Incorporation and standardization of biomarker techniques, and multi-biomarker strategies to guide selection of combination ICI approaches will facilitate expansion of the clinical benefit of immunotherapy to appropriate subgroups of patients with advanced cancer.

## Data Availability

Not applicable.

## Abbreviations

ICI: Immune checkpoint inhibitors
TME: Tumor microenvironment
FDA: Food and Drug Administration
CTLA-4: Cytolytic T lymphocyte antigen 4
PD-1: Programmed cell death protein
TMB: Tumor mutational burden
MSI: Microsatellite Instability
GEP: Gene expression profiling
IHC: Immunohistochemistry
IF: Immunofluorescence
TIL: Tumor infiltrating lymphocytes
TCR: T cell receptor
CPS: Combined positive score
TPS: Tumor proportion score
NSCLC: Non-small cell lung cancer
MMR: Mismatch repair
HLA: Human leukocyte antigen
PCR: Polymerase chain reaction
NGS: Next-generation sequencing
WES: Whole exome sequencing
PFS: Progression-free survival
IFN: Interferon
ctDNA: Circulating tumor DNA
MDSC: Myeloid derived suppressor cells
TAM: Tumor associated macrophages
scRNA-seq: Single-cell RNA sequencing
CAF: Carcinoma-associated fibroblasts
IDSC: Investigational Drug Steering Committee
